# Transactions of the College of Physicians of Philadelphia

**Published:** 1845-11

**Authors:** 


### *Transactions of the College of Physicians of Philadelphia.*

*Stated Meeting, April 1st*, 1845.—Dr. Pepper read the following account of a case of Peritonitis, *with perforation of the appendicula vermiformis*, caused, apparently, by the impaction in the latter of a grape seed.

Mrs. H——, aged 45, the mother of a numerous family, had enjoyed uninterrupted good health up to the latter part of January, 1845, when, for the first time, she complained of some lassitude, and occasional uneasy sensations in the right iliac region. From the 2d of February up to the 5th of the same month, her bowels had been constipated, and she accordingly took a dose of oleum ricini, followed by an enema of senna and sulph. magnesiæ—without, however, producing the desired effect. On Thursday, the 6th, about 3 A. M., she was *suddenly* seized with severe pain in the right iliac fossa, attended with great nausea and vomiting, prostration of strength, and collapsed countenance.

The patient was now for the first time confined to her bed, and treated by a charlatan, under whose care she continued up to February the 8th, without any important change in her symptoms.

Dr. E. Peace was now requested to visit the patient, and found her in the following condition:—pulse 130, and feeble; extremities warm; features collapsed; skin moist, but of a natural temperature; total loss of appetite, but no vomiting; abdomen tympanitic. She complained of extreme pain about the umbilicus, and also of great tenderness in the right iliac fossa. An ounce of oleum ricini, combined with sulph. morph. gr. ss., was now administered, but could not be retained; and a similar dose was repeated several hours after. The patient was also placed in a warm bath, and saline enemata were freely used, without, however, evacuating her bowels, which had now been in a constipated condition for the last five days.

Whilst under the above treatment, the tympanitis and pain diminished, but the patient gradually became more feeble, and expired the following morning, February the 9th, at 5 o'clock.

*Post-mortem examination* the following day. Present, Drs. Peace and Garrison.—Abdominal muscles covered with a thick layer of adipous matter. On opening the peritoneum, there was an escape of fœtid gas, having a somewhat gangrenous odour; and the serous membrane throughout presented evident traces of recent inflammation. In the right iliac fossa, several convolutions of the small intestines were united to each other, as also to the caput-coli, by means of strong, false membranes; and, at this part, the peritoneum was bathed with pus. The appendicula vermiformis was greatly thickened, and adhered, throughout its whole extent, to the lower part of the ileum; near its free extremity, there was an ulcerated opening, about one-fourth of an inch in diameter.

The lower part of the ileum, in connection with the cœcum, was now removed for more minute inspection. On laying open the appendix, it was found to contain, at its perforated extremity, a small mass of indurated fæcal matter, in one end of which was imbedded a large grape-seed; its mucous membrane was black, and in a gangrenous condition—whilst the other coats were softened and greatly thickened. The ileum, as also the caput-coli, were apparently perfectly healthy. The pelvis contained several ounces of a thin brownish fluid, having a somewhat fæcal odour. Intestines distended with flatus; other viscera not examined.

From the previous history, it is highly probable that the grape-seed was the exciting cause of the inflammation, which ultimately led to ulceration and fatal perforation of the appendix. Such cases can no longer be considered as extremely rare, since numerous instances of a somewhat similar character are now to be found on record. In the Medical Examiner, vol. i. p. 655, is reported a case of fatal peritonitis from two perforations of the appendix, caused, most probably, by "a gritty, brown substance, about the size of a bean." In the same journal, vol. iii. p. 580, may be found a case of chronic inflammation of the appendix, which ultimately terminated in a fistulous opening through the abdominal parietes. In this case, the appendix was thickened and dilated, and the shell of a hazel-nut was impacted in its middle portion.

Dr. Morris read the following account of two cases of hæmoptysis, occurring in infants of three months.

In the month of January last, I was called to render medical aid to the child of Mr. Hastings, aged three months. It was a male, one of twins, of large size, and fine health up to the time of its attack. The mother, a woman of unusual intelligence and energy, reported the child to be labouring under an attack of colic, with diarrhœa of some days' standing. The discharges were frequent, green, and slimy. There was much heat of skin and thirst. The abdomen was flaccid, and the face anxious. The child cried much, and the sound was plaintive. The usual prescriptions in such cases were made: warm poultices to the bowels; small doses of calomel and castor oil. By these means, the condition of the child was improved, though the heat of skin and febrile disturbance still continued. About the third day of my attendance, the mother showed me her collar, which was smeared with blood, that, she assured me, had been discharged from the mouth of her infant. I at once ascribed it to some ulceration of her nipple, for which I searched carefully, but without being able to detect the slightest solution of continuity. The gums and throat of the child were next examined, and an ulcer was found near the isthmus faucium, to which, though small, the issue of blood was supposed to be owing. The following day she exhibited a handkerchief much stained with blood, of a pink hue, which had been discharged again by the child. Dr. Meigs was now requested to see the infant, and his suspicions were so strong that the blood was derived from the breast of the mother, and vomited by the child, that he could not be persuaded to the contrary till he had examined the nipple with a magnifier, and applied smart friction with a cambric handkerchief. The respiration of the child did not deviate from the natural state sufficiently to attract our attention, though we visited it often, and examined it anxiously; nor was there, at any time, the slightest cough. After lingering a few days — the diarrhœa varying in degree — it sank into coma, followed by convulsions and death. The autopsic examination, made by my friend, Dr. Sargent, Resident Physician to the Pennsylvania Hospital, was as follows:

Exterior aspect of body pale; hands and feet of a blueish hue; similar colour of all the dependent parts of the body and limbs; very slight, if any, emaciation. The mouth and nose contained a frothy, reddish-white fluid, which escaped freely from these cavities when placed in a dependent position.

Brain not examined—nor posterior part of mouth and fauces. Larynx perfectly healthy.

The mucous membrane of the trachea and of the bronchial tubes, as far as their fourth or fifth ramifications, was reddened, and, in the bronchial tubes, slightly thickened, but in both entirely free from false membrane, or viscid secretion of any kind. The inferior portion of both lungs soft and crepitant, but containing abundance of a colourless serous fluid.

The lower lobe of the left lung was of a vivid red colour; contained a very small quantity of air, and but little serum; consistence of its tissue not materially diminished. The right lung was of the same bright red colour in its middle and lower lobes; the consistence of both these lobes was diminished, particularly of the middle; and the greater portion of this lobe sank when placed in water of medium temperature. There was no appearance of pus, nor of lobular discolouration or induration. No adhesion between the opposed pleural surfaces of either side; nor any effusion of lymph or serum in either pleural cavity. Both lungs were expanded. No tubercles in the lungs, or in the bronchial tubes.

The heart was of a healthy appearance; foramen ovale open.

The abdominal viscera were all carefully examined, but no lesion of any kind could I detect.

Within a week after, the surviving child was taken with symptoms precisely similar. Guided by the light derived from the examination of the preceding case, our attention was now turned to the chest. With great difficulty, owing to the extreme restlessness of the infant, we were enabled to detect dulness, upon percussion, of the posterior part of the lower lobe of the right lung, with slight mucous rattle. The quantity of blood dlscharged, though less than that in the preceding case, was considerable; and in this instance I saw it in the mouth of the child, frothy, and mixed with mucus. Small cups were applied to the back of the child, and two ounces of blood taken. Calomel, in minute doses, was given to act upon the diseased secretions of the liver; and, in a day or two, our patient was convalescent.

The only notice of this symptom I have met with, is in the work of Rilliet and Barthez, who speak of their experience as confirmatory of that of Dr. Gerhard, who says he has never observed, in the pneumonia of infants, the bloody sputa attendant upon the disease when it occurs in the adult. They refer to the testimony of Valliex, who reports having met with a discharge of bloody mucus in the pneumonia of infants; a circumstance, however, of which they are evidently disposed to question the truth.

Dr. Ashmead related the case of a patient under his care, to whom an ordinary dose of calomel was given as a purgative, and followed by a dose of magnesia—who, soon after taking the calomel, was seized with symptoms similar to those resulting from poisoning with corrosive sublimate. He was treated by the usual antidotes and remedies indicated in such cases, and recovered. There was no reason to suspect that the calomel given in this case was an impure article, or that there was any idiosyncrasy of constitution, as the patient had previously taken calomel without any unpleasant symptoms following.

Dr. Morris presented the following account of a *case of deat from extensive disease of the stomach,* unattended, during the lif time of the patient, with any symptoms indicative of its existence.

The case is related by Dr. J. M. Paul, who was the physician in attendance.

B. G., aged 59 years, a gentleman of a nervo-sanguineous temperament; active, industrious, and temperate in his habits; had enjoyed perfect and uninterrupted health, with the exception of slight and occasional attacks of diarrhœa, for the last year of his life.

On the evening of Wednesday, the 19th of March last, he returned home from his place of business, much fatigued in body and in mind; complaining of weariness and aching of the limbs, chilliness, sore throat, headach, and some febrile excitement. His wife persuaded him to bathe his feet in hot water, with the addition of mustard, and to take some magnesia and Epsom salts, which he did, and retired to bed. During the night, he experienced great restlessness, pain in the head, nausea, and some vomiting of bilious fluid, with a sensation of distress at the epigastrium; but these symptoms were partially relieved by the operation of the purgative medicines he had taken, and towards morning he felt himself better, and slept.

I first visited him about 11 o'clock on the Thursday morning ensuing, and found him labouring under a slight fever, some headach, oppression at the stomach—with an obscure tenderness on pressure. The epigastrium yielded, on percussion, a tympanitic sound; the tongue was coated and white; the pulse was moderately full, but regular. He was directed to keep perfectly quiet; to take, occasionally, a mixture of the citrate of potash with sp. nitri dulcis; to apply a sinapism over the epigastrium, followed by a mush poultice; and to use ice, and mucilaginous drinks. In the evening he took six grains of the blue mass, and repeated the warm foot bath.

On Friday, the 21st, he felt so well, as to be able to return to his place of business, and there he continued until late in the evening, attending to his affairs. In the evening, he returned home much fatigued—complaining of chilliness, headach, &c. Bathed his feet in hot water and mustard, took a cup of weak tea, and retired to his bed. His wife gave him, in the night, some magnesia, which operated well, but he had a restless night; some wandering, headach, and a sense of weight or obstruction at the pit of the stomach; flatulence, and occasional nausea, but no vomiting.

On the morning of the 22d, I found him feverish, with a pulse full and active; tongue coated with a thin white covering; and complaining of pain in the head: his bowels had been freely moved. I took away xvj. oz. of blood, which relieved his head; it was buffy and slightly cupped; reapplied the mustard over the stomach, followed by poultices of mush, and directed him a mixture of the bicarbonate of potash, with a little camphor water, to relieve his flatulence. Ice to be taken freely; cold applications to the head; mucilaginous drinks, &c.

These remedies gave him comfort, and in the evening he was better, and expressed himself much relieved.

At night he took six grains of the blue pill, and repeated the foot bath; continued the ice, poultices, and mucilaginous drinks.

On Sunday, the 23d, he continued much the same; his symptoms were, however, milder. The same treatment was continued.

On Monday morning, the 24th, he appeared convalescent; expressed himself as decidedly better; the fever had subsided; pulse good and regular; head relieved; stomach more comfortable, though still exhibiting some tenderness on pressure. There was considerable muscular prostration, but the general aspect of the patient was good and favourable. There was nothing present to indicate danger; the patient, indeed, thought himself almost well. During my visit, I carefully examined the epigastric and abdominal regions, and finding that pressure still gave him pain, by way of precaution, I proposed that some twenty leeches should be applied over the painful part, to be succeeded by a large poultice of corn meal; to this he acceded. The leeches were applied, but took a long time in taking hold; this caused the patient great fatigue, from the confined position to which he was necessarily subjected. A small quantity of blood was drawn by the leeches; after they fell off, he sank into a sleep, which lasted for half an hour: when he suddenly awoke; jumped out of bed to go to the chair, as had been his custom; reached the middle of his chamber; became faint, and was near falling, when he was assisted to bed with difficulty.

I saw him half an hour after this occurrence; found him utterly prostrated; breathing rapidly, and with a pulse of 150 to 160 a minute. I immediately gave him freely of brandy, aromatic spirits of ammonia, and hot nourishment; hot bricks were applied to the feet, with sinapisms, &c.; but without much, if any, effect. Pulse continued rapid and feeble; prostration extreme.

At my request, Dr. Caspar Morris was called in consultation. He considered the patient as alarmingly ill; approved of the stimulants, and suggested the application of a blister over the epigastrium, cold to the head, and ice to be taken internally, and wine whey to be substituted for the brandy. This was at 2 o'clock, P. M. I continued with him, and Dr. Morris saw him again in a few hours. The indications of sinking continued, and the case was evidently hopeless. The mind of the patient was collected and clear; had no idea of his danger, and could not feel that he was ill. He died about 6½ o'clock, apparently in a gentle sleep.

*Autopsy.* Present, Drs. Paul, C. Morris, and J. Neill.—The external appearances of the body generally, were natural; some lividity existed about the extremities. The abdomen was opened through the linea alba: the adipose tissue was one inch in thickness. On displaying the viscera in their natural position, there was exhibited a slight adhesion of the omentum majus to the parietes, at the epigastric region. Large patches of coagulated lymph were found upon the great omentum, which extended to the left hypochondriac region, completely covering the anterior face of the stomach, and a portion of the spleen. The superior face of the liver was also found to be coated by a lamina of coagulated lymph, which could be removed in masses. The peritoneum of the parietes of the abdomen exhibited a venous injection, and the cavity contained a pint or more of serum.

The stomach was removed, and found to be exceedingly heavy, weighing, probably, one and a half pounds; it contained a thick fluid of a light brown colour. Its walls were about half an inch in thickness—the increase being principally in the cellular and mucous coats. The cellular coat, near the cardia, was filled with coagulated lymph, which became less firm near the middle; at the pylorus, the cellular coat was distended to the same thickness with serum. The mucous coat exhibited great changes in structure and appearance—being much thickened, and very firm: its hue was florid—the colour being deeper at the greater curvature. Its surface presented a very rough, granular formation, each projection being about the size of a grain of coffee, or larger. These were dense and unyielding to the touch, and more vascular than the surrounding tissue; they were more numerous at the greater curvature.

The liver presented no unusual appearance in its glandular structure.

A section of the upper part of the jejunum, and a section of the ileum, were examined; but presented no appearance of disease in any of their coats, except the injected peritoneum.

The examination was interesting in many respects. It proved that, with such altered structures, the case was beyond the reach of medicine. It presented traces of great inflammation, with little accompanying pain. It presented abnormal structures, which must have taken time to form, during, apparently, the enjoyment of good health. It shows that extensive disorganizations may take place in some tissues, without the occurrence of any symptoms by which their existence is revealed to either the patient or his physician.

